# Mapping of *Aegilops speltoides* derived leaf rust and stripe rust resistance genes using 35K SNP array

**DOI:** 10.1186/s12863-024-01247-5

**Published:** 2024-07-15

**Authors:** Balihar Kaur, Bukke Kutti Bai, Guriqbal Singh Dhillon, Jaspal Kaur, Achla Sharma, Puja Srivastava, Parveen Chhuneja, Satinder Kaur

**Affiliations:** 1 School of Agricultural Biotechnology, Punjab Agricultural University, Ludhiana, 141004 India; 2 University of Idaho, Moscow, USA; 3 Department of Plant Breeding and Genetics, Punjab Agricultural University, Ludhiana, 141004 India

**Keywords:** Leaf rust, Stripe rust, *Aegilops speltoides*, Bulk segregant analysis, Genotyping, Linkage mapping

## Abstract

Wheat is an essential food commodity cultivated throughout the world. However, this crop faces continuous threats from fungal pathogens, leaf rust (LR) and stripe rust (YR). To continue feeding the growing population, these major destructors of wheat must be effectively countered by enhancing the genetic diversity of cultivated germplasm. In this study, an introgression line with hexaploid background (IL^*sp3603*^) carrying resistance against *Pt* pathotypes 77−5 (121R63-1), 77−9 (121R60-1) and *Pst* pathotypes 46S119 (46E159), 110S119 (110E159), 238S119 (238E159) was developed from donor wheat wild progenitor, *Aegilops speltoides* acc pau 3603. To understand the genetic basis of resistance and map these genes (named *Lr*^*sp3603*^ and *Yr*^*sp3603*^), inheritance studies were carried out in F_6_ and F_7_ mapping population, developed by crossing IL^*sp3603*^ with LR and YR susceptible cultivar WL711, which revealed a monogenic (single gene) inheritance pattern for each of these traits. Bulk segregant analysis combined with 35 K Axiom SNP array genotyping mapped both genes as separate entities on the short arm of chromosome 6B. A genetic linkage map, comprising five markers, 1 SNP, 1 PLUG and three gene based SSRs, covered a genetic distance of 12.65 cM. *Lr*^*sp3603*^ was flanked by markers *Tag-SSR14* (located proximally at 2.42 cM) and SNP *AX-94542331* (at 3.28 cM) while *Yr*^*sp3603*^ was mapped at one end closest to *AX-94542331* at 6.62 cM distance. Functional annotation of *Lr*^*sp3603*^ target region (∼ 1 Mbp) revealed 10 gene IDs associated with disease resistance mechanisms including three encoding typical R gene domains.

**Supplementary Information:**

The online version contains supplementary material available at 10.1186/s12863-024-01247-5.

## Background

Leaf rust (LR) (*Puccinia triticina* Eriks, *Pt*) and stripe rust (YR) (*Puccinia striiformis f. sp. tritici*, *Pst*) stand as the two most pervasive foliar diseases significantly impacting global wheat yield. With nearly one-third of the world’s population reliant on wheat as a source of staple carbohydrates and protein [1] and the ever-increasing population on earth (https://www.un.org), it is essential to confront these persistent fungal diseases to sustain food security. The LR outbreaks have been reported to result in yield losses up to 50%, while YR has been documented to incur higher yield losses reaching up to 70%, due to their detrimental effects on the photosynthetic and grain-filling capacity of the crop [2, 3]. The degree of damage, however, is also influenced by the time of infection, environmental conditions, cultivar susceptibility and aggressiveness of the pathogen [1, 4, 5]. India, being one of the leading wheat-producing countries, faces a high incidence of these rusts, particularly in the Northern parts known as the breadbasket of India, due to suitable environmental conditions for pathogens [5–7]. The success of the green revolution with the breeding of superior yielding disease-resistant varieties has undeniably resulted in a significant boost in agricultural productivity. However, wheat has succumbed to domestication bottlenecks because of narrow genetic diversity in cultivated germplasm, thereby making it vulnerable to rising biotic and abiotic threats [8, 9]. Therefore, improvement of germplasm through genetic reshuffling is imperative to confront the evolving nature of rusts.

Wild germplasm contains a vast array of genetic diversity that can be harnessed to enhance crop resilience [10]. Incorporation of genetic resistance through wild introgressions offers a more environmentally friendly, cost-effective, and reliable strategy for mitigation of fungal infestation in comparison to chemical control with fungicides [11]. Numerous LR and YR resistance genes have been identified from different wild species such as *Lr53/Yr35, Lr64, Yr15, Yr36* from *Triticum dicoccoides* [12–15], *Lr18, Lr50* from *Triticum timopheevii* [16, 17], *Lr9, Lr76/Yr70* from *Aegilops umbellulata* [18, 19], *Lr54/Yr37* from *Aegilops kotschyi* [20], *Lr56, Yr38* from *Aegilops sharonensis* [21], *Lr57/Yr40* from *Aegilops geniculata* [22], *Lr59, LrAp* from *Aegilops peregrina* [23, 24], *Lrtri, Yrtri* from *Aegilops triuncialis* [25], *LrAc, YrAc* from *Aegilops caudata* [26] and many more [27].

*Aegilops speltoides* (2n = 14, SS), a close diploid relative of the B genome in the secondary gene pool of wheat, is an important wild species harbouring a plethora of genes for wheat improvement [28]. It has been previously reported to contribute resistance against several biotic stresses like leaf rust (*Lr28, Lr35, Lr36, Lr47, Lr51, Lr65, Lr66*), stem rust (*Sr32, Sr39, Sr47, Sr54*), powdery mildew (*Pm1d, Pm12, Pm32, Pm53*) and green bug (*Gb5*) [29–35].

Among the wild germplasm collection maintained by Punjab Agricultural University (PAU), *Ae. speltoides* (SS) accession pau 3603 was found resistant against prevalent LR and YR pathotypes in Punjab. The resistance against these two rusts has been transferred into hexaploid wheat background and an introgression line named IL^*sp3603*^ with resistance to both rusts has been developed. In the present experiment, the IL^*sp3603*^ was crossed with LR and YR susceptible wheat, WL711 and a mapping population was developed to understand the genetics of introgressed resistance and mapping of genomic regions harbouring rust resistance using 35K SNP array.

## Materials and methods

### Introgression line IL^*sp3603*^ and mapping population

The plant material consists of LR and YR resistant introgression line IL^*sp3603*^, LR and YR susceptible wheat line WL711 and 152 F_2_:F_6_ mapping population developed by their intercrossing. Introgression line IL^*sp3603*^ was developed at the School of Agricultural Biotechnology, PAU, Ludhiana, by initially crossing LR and YR resistant diploid S genome wheat progenitor, *Ae. speltoides* accession pau 3603 (2n = 14) with tetraploid durum wheat, PDW233 (2n = 28). Thereafter, the resulting progeny was crossed and backcrossed with LR and YR susceptible hexaploid wheat, PBW621 (2n = 42), followed by continued selfing for five generations. The introgression line IL^*sp3603*^, having resistance to LR and YR, and chromosome number 2n = 42 was selected by screening selfed progenies against LR and YR in each generation. The IL^*sp3603*^ × WL711 cross was made to develop F_6_ and F_7_ RILs to understand the genetics of LR and YR resistance transferred from *Ae. speltoides* and their mapping onto specific chromosomes (Fig.S1).

### Rust screening

#### Seedling stage

Seedlings of parental lines and mapping population were tested against prevalent *Puccinia triticina* (*Pt*) pathotype 77−9 (121R60-1) having avirulence/virulence formula: *Lr2a, Lr2b, Lr2c, Lr9, Lr19, Lr24, Lr25, Lr28, Lr32, Lr39, Lr42, Lr45, Lr47*/ *Lr1, Lr3, Lr10, Lr11, Lr12, Lr13, Lr14a, Lr14b, Lr14ab, Lr15, Lr16, Lr17a, Lr17b, Lr18, Lr20, Lr21, Lr22a, Lr22b, Lr23, Lr26, Lr27 + 31, Lr30, Lr33, Lr34, Lr35, Lr36, Lr37, Lr38, Lr44, Lr46, Lr48, Lr49* [36]. Seeds were sown in rows in plastic boxes (25 cm × 10 cm), with 8 rows/box and 10–12 seeds per row, with the first row as susceptible check WL711 in each box. For achieving uniform spread and the desired concentration of urediniospores, one week old seedlings were inoculated with a mixture of pathotype 77−9 and talcum powder, incubated overnight under 100% humidity in the dark and then shifted to a glasshouse maintained at 18–20 °C. Leaf rust infection types (ITs) were scored 14 days after inoculation following modified Stakman 0–4 scale [37].

### Adult plant stage

At the adult plant stage, parental lines and mapping population were planted in two separate sets and evaluated for LR and YR, respectively for two cropping seasons, 2021-22 (F_6_) and 2022-23 (F_7_). A mixture of *Puccinia striiformis* (*Pst*) pathotypes, 46S119 (46E159), 110S119 (110E159) and 238S119 (238E159) was used for testing against YR while a mixture of *Pt* pathotypes, 77−5 (121R63-1) and 77−9 (121R60-1) was used to screen against LR (Table S1). Scoring against both rusts was executed by assessing the percentage of leaf area covered, with ITs assigned following modified Cobb’s scale [38].

### DNA extraction and 35K SNP array genotyping

Genomic DNA was extracted from young leaves using a modified CTAB protocol [39] and the concentration and purity of DNA were evaluated on NanoDrop Spectrophotometer. For bulked segregant analysis (BSA), two sets of contrasting bulks were prepared against both LR and YR. Twenty homozygous resistant (HR) and 20 homozygous susceptible (HS) F_6_ progenies were selected each for LR and YR. Single plant DNA from 10 selected F_6_ progenies was pooled, and four bulks were formed, each for LR (LrRB1, LrRB2, LrSB1, LrSB2) and YR (YrRB1, YrRB2, YrSB1, YrSB2). Genotyping of these bulks and parental lines was performed using 35K Axiom wheat breeder’s SNP array (AFFYMETRIX) (Fig. S2). Raw data was processed by filtering SNPs with missing data points or unassigned chromosomes. In the finally selected data, polymorphic SNPs were identified between contrasting parental lines and bulks.

### Primer designing and linkage mapping

Axiom IDs associated with potentially linked SNPs were used for designing KASP assays using the pipeline Polymarker [40]. Two allele-specific forward primers (each labelled with HEX 5-GAAGGTCGGAGTCAACGGATT-3 and FAM 5-GAAGGTGACCAAGTTCATGCT-3 sequences), and a common reverse primer were synthesized. PCR assays were carried out in 4 µl reaction volume, containing 2 µl genomic DNA (30 ng/µl), 1.96 µl 2X KASP reaction mix and 0.054 µl KASP assay mix (containing 3 primers) in 384-well plate on thermocycler (Applied Biosystems Veriti) at 95 °C for 15 min; 10 touchdown cycles of 95 °C for 10 s, 61 –57 °C for 1 min (0.6 °C drop per min); 25 cycles at 95 °C for 20 s and annealing at 57 °C for 1 min. Fluorescence intensity was recorded by Tecan infinite F200 PRO plate reader, and results were imported to Kluster Caller software (LGC Biosearch Technologies v3.4.1.36).

To saturate the genetic region localized by SNP markers, we also used some already reported SSR markers [41], PCR-based landmark unique genes (PLUG) [42] and also designed new SSRs from the targeted region using the MIcroSAtellite identification tool, MISA v2.1 [43]. These markers were amplified in 10 µl PCR reaction mixture having 2 µl (60ng) DNA, 4 µl 2X EmeraldAmp PCR Mastermix, and 1 µl (0.01mM) forward and reverse primer each. PCR amplification was carried out in a 96-well thermocycler for 35 cycles at 94 °C for 1 min, 53–61 °C for 1 min, 72 °C for 2 min. PCR products were separated on 3.0-3.5% agarose gel. Polymorphic SNPs, SSRs and PLUG markers were amplified onto the entire mapping population.

### Homoeologue confirmation for leaf rust and stripe rust resistance

To determine the presence of linked markers on chromosome 6B, these markers were amplified on nullisomic-tetrasomic stocks, *CS-N6A-T6D, CS-N6B-T6A, CS-N6D-T6A* and *CS-N6D-T6B* available in cultivar Chinese Spring background (acquired from Wheat Genetics Resource Center, Kansas State University, Manhattan).

### Statistical analysis

Chi-square analysis was carried out to determine the goodness of fit of observed genotypic and phenotypic data with the expected genetic ratios using the following formula:

$${{\rm{\chi }}^{\rm{2}}}\,{\rm{ = }}\,\sum {{{\left( {{\rm{O - E}}} \right)}^{{\rm{2}}}}{\rm{/}}{\rm{E}}}$$

where χ^2^ = chi-squared, test statistic, O = observed phenotype in each category, E = expected phenotype in each category. Linkage analysis was conducted using QTL ICi mapping software v4.2. Kosambi mapping function was used to calculate recombination frequencies and estimate map distances in centimorgans (cM). A LOD score value of 3 was set, and the ‘GROUPING’, ‘ORDERING’ and ‘RIPPLING’ commands were carried out sequentially to yield the final marker order. Following, linkage map was constructed using the Microsoft excel software, MapDrawJZ-v2 [44].

### Physical map construction and target annotation

Primer sequences corresponding to mapped markers were used for extracting physical positions through blast search against Chinese Spring reference genome assembly (IWGSC RefSeq v2.1). The DNA sequence within the target region, bordered by flanking markers, was further used for retrieving gene IDs.

## Results

### Rust response against LR and YR pathotypes

#### Leaf rust

**Fig. 1:**
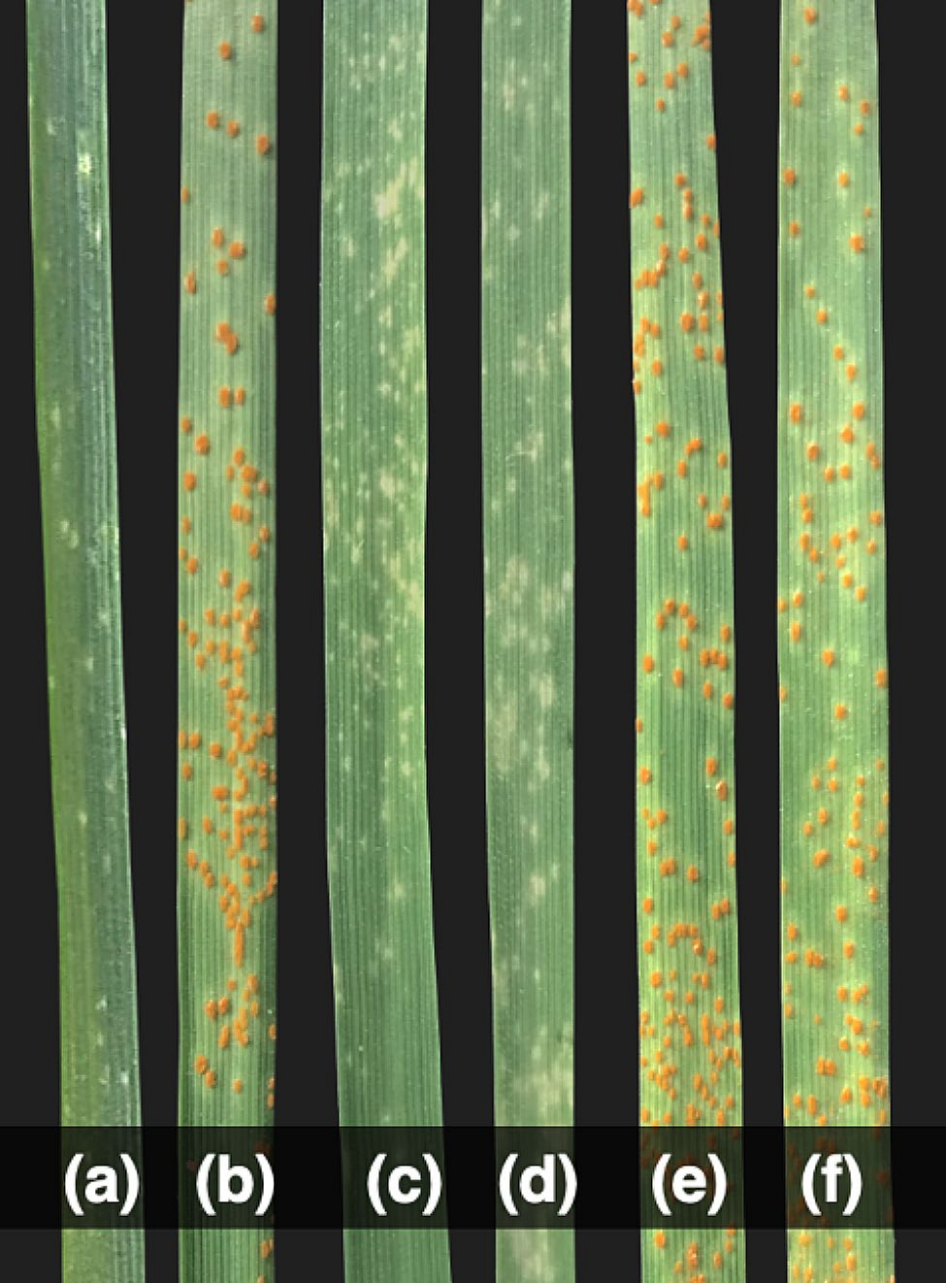
Infection type against leaf rust pathotype 77 − 9 (121R60-1) at seedling stage (**a**) IL^*sp3603*^ (**b**) WL711 (**c**, **d**) resistant F_6_ progenies (**e**, **f**) susceptible F_6_ progenies

At the seedling stage, IL^*sp3603*^ was resistant (IT=;) to *Pt* pathotype 77 − 9 while WL711 was susceptible (IT = 3) (Fig. 1). Similarly, at the adult plant stage, IL^*sp3603*^ had only traces (TR) of LR severity while WL711 was susceptible with a severity of 60–80 S. In F_6_, there were 77 Homozygous Resistant (HR), 14 Segregating (Seg) and 61 Homozygous Susceptible (HS) progenies with χ² _(1.875:0.25:1.875)_ = 3.51, p-value = 0.173 while in F_7_, there were 82 HR: 7Seg: 63HS progenies (χ² _(1.9375:0.125:1.9375)_ = 3.55, p-value = 0.169) at both the seedling and adult plant stages. The LR data at both the growth stages indicated the inheritance of a single LR resistance gene, effective throughout the life of the plant. This LR resistance gene is an all-stage resistance gene and is temporarily designated as *Lr*^*sp3603*^ (Table 1).

**Table 1:** Segregation of F_6_ and F_7_ progenies from cross IL^*sp3603*^× WL711 as HR (Homozygous Resistant), Seg (Segregating) and HS (Homozygous Susceptible)

Generation	No. of lines				χ²	p-value (*p* < 0.05)
	HR	Seg	HS	Total		
*Leaf rust*						
F_6_	77	14	61	152	3.51 (1.875:0.25:1.875)	0.173
F_7_	82	7	63	152	3.55 (1.9375:0.125:1.9375)	0.169
*Stripe rust*						
F_6_	77	13	62	152	2.55 (1.875:0.25:1.875)	0.279
F_7_	85	5	62	152	3.61 (1.9375:0.125:1.9375)	0.164

### Stripe rust

**Fig. 2:**
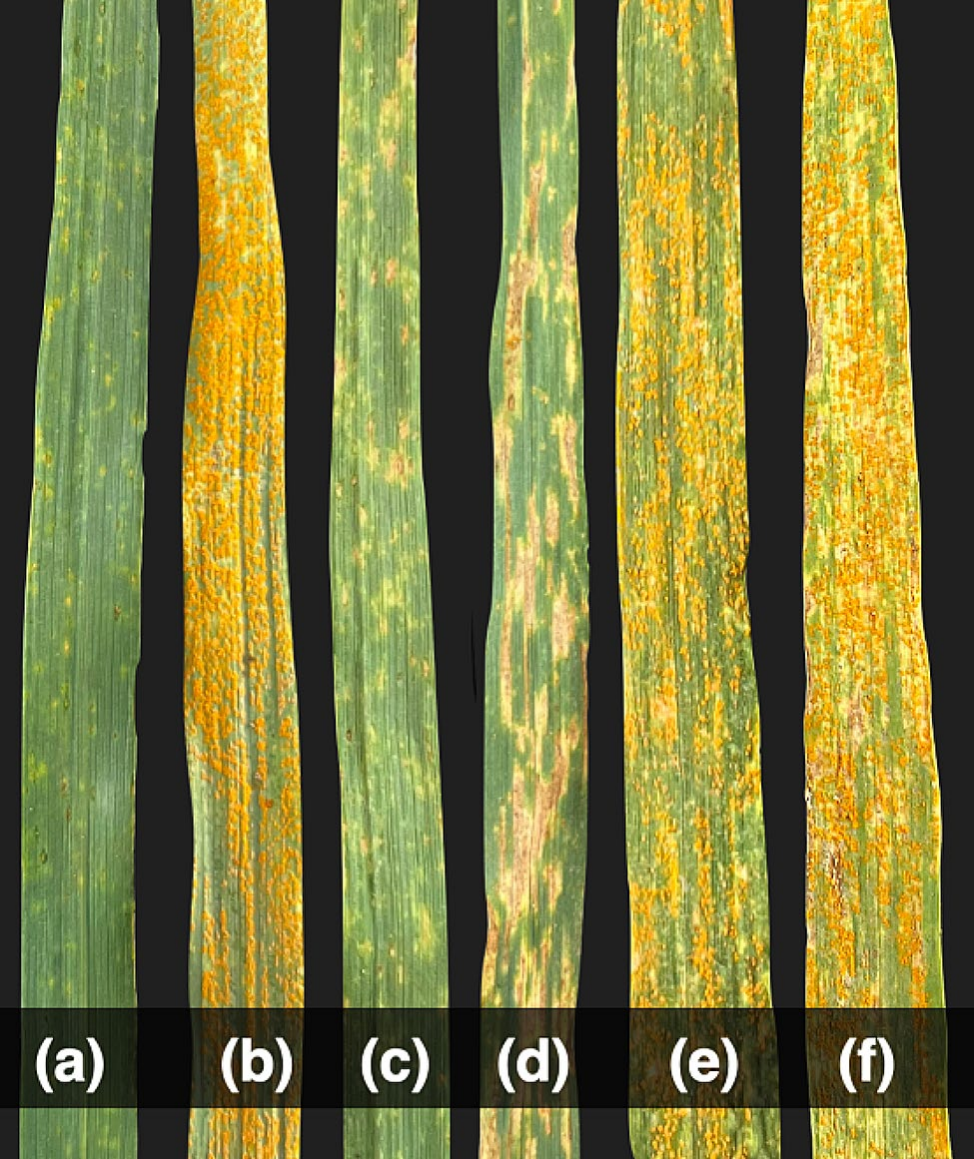
Stripe rust response recorded as disease severity against mixture of yellow rust pathotypes at adult plant stage: (**a**) IL^*sp3603*^ (**b**) WL711 (**c**, **d**) resistant F_6_ progenies (**e**, **f**) susceptible F_6_ progenies

IL^*sp3603*^ was resistant to YR at the seedling stage with IT of “ ; ”, and at the adult plant stage, it showed YR severity of traces (TR). WL711 was susceptible with IT = 3 at the seedling stage and YR severity of 80S at the adult plant stage (Fig. 2). Out of 152 F_6_ progenies, 77 were HR, 13 were found to be Seg, while the remaining 62 were HS with χ² _(1.875:0.25:1.875)_ = 2.55, p-value = 0.279. In F_7_ generation, there were 85 HR: 5Seg: 62HS progenies (χ² _(1.9375:0.125:1.9375)_ = 3.61, p-value = 0.164). Segregation of progenies at seedling and adult plant stages at F_6_ and F_7_ generation revealed that the YR segregation pattern also adhered to the monogenic inheritance of an all-stage resistance gene (Table 1). This YR resistance gene has been temporarily designated as *Yr*^*sp3603*^.

### Association between *Lr*^***sp3603***^ and *Yr*^*sp3603*^

LR and YR scores were compared in F_7_ progenies to determine the association between *Lr*^*sp3603*^ and *Yr*^*sp3603*^. Of the total 152 F_7_ progenies, 130 (∼ 86%) showed similar reaction for LR and YR, meaning progenies were either HR or Seg or HS for both LR and YR, and the remaining 22 progenies (∼ 14%) showed contrasting phenotype, either resistant for LR and susceptible for YR or vice versa, suggesting recombination between *Lr*^*sp3603*^ and *Yr*^*sp3603*^ genes. This implies that these two genes exist as separate entities though closely located on the same chromosome, with a genetic distance of approximately 14 cM (Table 2).

**Table 2:** Two-way table showing comparison of number of F_7_ homozygous resistant (HR), segregating (Seg) and homozygous susceptible (HS) progenies for leaf rust and stripe rust

	Leaf rust response				
**Yellow rust response**		**HR**	**Seg**	**HS**	**Total**
	**HR**	73	3	9	85
	**Seg**	0	4	1	5
	**HS**	9	0	53	62
	**Total**	82	7	63	152

### Mapping through BSA-35K SNP data analysis

#### Leaf rust

**Fig. 3:**
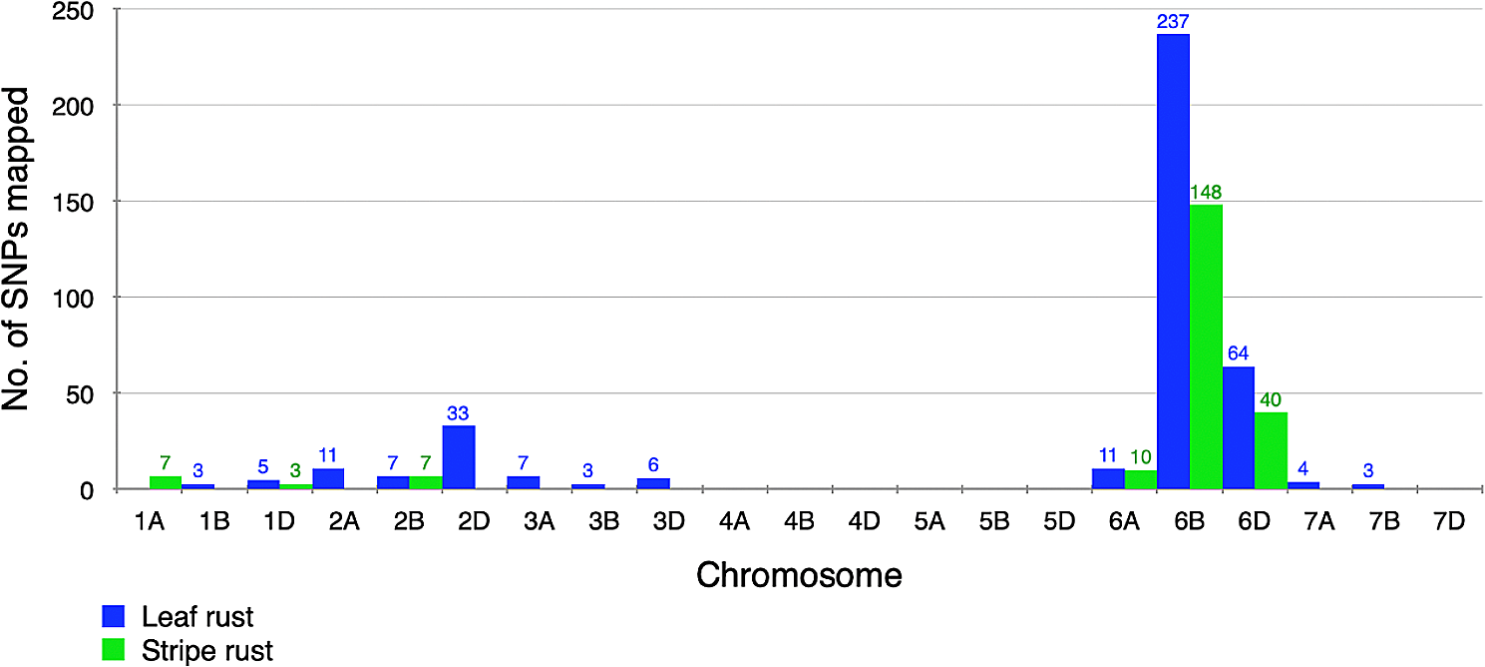
Distribution of polymorphic SNPs from 35K SNP array data on 21 wheat chromosomes. Blue color represents SNPs polymorphic between leaf rust resistant and susceptible bulks while green color represents SNPs polymorphic between stripe rust resistant and susceptible bulks, both the bulks generated using F_6_ progenies of cross, IL^*sp3603*^ and WL711

The parents, IL^*sp3603*^ and WL711 along with LR bulks (LrRB1, LrRB2, LrSB1, LrSB2) were genotyped using 35K Wheat Breeders’ SNP array. After filtering, 24,693 SNPs spanning throughout the genome were retained of which 833 SNPs were polymorphic between contrasting parents and bulks. The highest proportion of these polymorphic SNPs (∼ 60%) was on chromosome 6B, followed by 16.2% on chromosome 6D and 8.4% on chromosome 2D (Fig. 3).

### Stripe rust

Similarly, independent processing of 35K SNP array data was carried out for parental lines and bulks prepared for YR (YrRB1, YrRB2, YrSB1, YrSB2), resulting in 26,985 filtered SNPs of which 474 SNPs were polymorphic between contrasting parental lines and bulks. These SNPs were distributed on chromosomes 1A, 1D, 2B, 6A, 6B and 6D, with the maximum number of the polymorphic SNPs lying on chromosome 6B (∼ 69%), followed by 6D (18.6%) and 6A (4.7%) (Fig. 3).

Notably, the maximum number of SNPs showing polymorphism between resistant and susceptible bulks, both for LR (237 SNPs) and YR (148 SNPs), were on chromosome 6B. Of these, 90 SNPs were common among LR and YR, suggesting the presence of both *Lr*^*sp3603*^ and *Yr*^*sp3603*^ genes in the vicinity of these SNPs on chromosome 6B (Fig. S3). A major number of closely positioned SNPs were lying on the short arm of chromosome 6B, spanning a physical interval of ∼ 35 Mbp. From these, 19 SNPs were selected and converted into PCR-based KASP assay for validation. Besides, this region was searched for already known markers from which 8 PLUG and 7 SSRs were selected (Table S2). Also, 15 new SSR markers were designed from the genic regions and were named “Tag-SSRs” (Table S3). All these SNP, SSR and PLUG markers were amplified on contrasting parents and bulks of which 10 markers including 4 SNP based KASP, 1 PLUG and 5 genic-SSRs (Tag-SSRs), were polymorphic.

### Mapping of *Lr*^*sp3603*^ and *Yr*^*sp3603*^

For linkage analysis and genetic map construction, 152 F_7_ progenies were amplified with 10 polymorphic markers of which 5 markers including one KASP (*AX-94542331*), one PLUG (*TNAC1674*) and three Tag-SSRs (*Tag-SSR10, Tag-SSR12* and *Tag-SSR14*) displayed segregation within the population. Marker *AX-94542331* showed distinct cluster formation and amplified the ‘T’ allele in IL^*sp3603*^ and resistant progenies, and the ‘C’ allele in WL711 & susceptible progenies (Fig. S4), with 5 recombinants observed with *Lr*^*sp3603*^ and 10 recombinants with *Yr*^*sp3603*^. The only polymorphic PLUG marker *TNAC1674* showed 14 and 29 recombinants with *Lr*^*sp3603*^ and *Yr*^*sp3603*^, respectively. It amplified the allele of 628 bp in IL^*sp3603*^ and resistant progenies, while an amplicon size of 600 bp was observed in WL711 and susceptible progenies *(*Fig. S5a). *Tag-SSR12* and *Tag-SSR14* had 4 and 19 recombinants with *Lr*^*sp3603*^ and *Yr*^*sp3603*^ genes, respectively. The former produced an amplicon size of 140 bp and 178 bp, while the latter amplified alleles of 146 bp and 165 bp in resistant and susceptible parents and progenies (Fig. S5b, c). The *Tag-SSR10* amplified a resistant allele of 175 bp and a susceptible allele of 160 bp (Fig. S5d) with 5 and 21 recombinants with *Lr*^*sp3603*^ and *Yr*^*sp3603*^ genes, respectively.

**Fig. 4:**
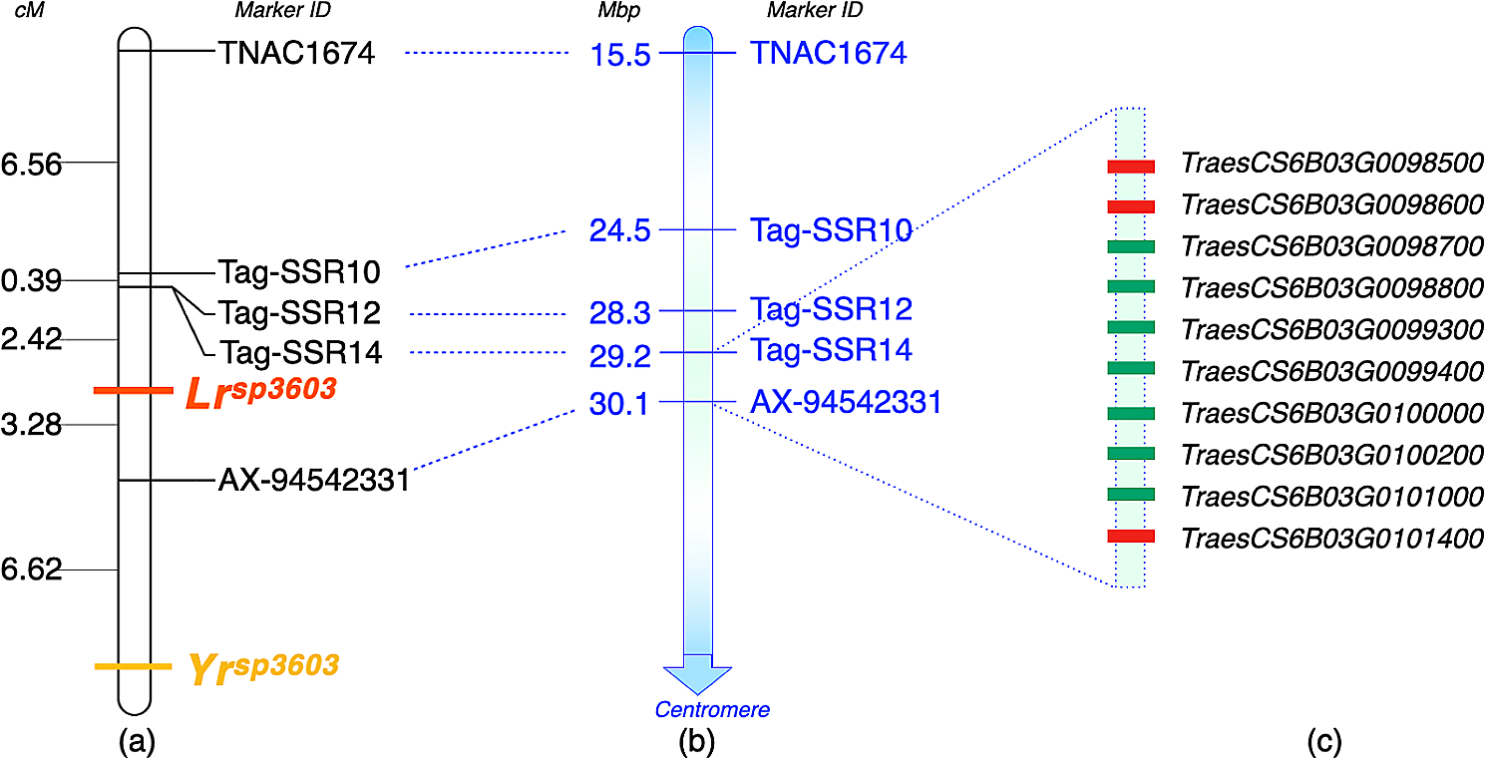
Comparison of (**a**) genetic map with (**b**) physical locations (IWGSC v2.1) of chromosome 6B and (**c**) corresponding annotations associated with disease resistance genes within the target region. Gene IDs marked in RED encode for NBS-LRR genes while GREEN represents other disease resistance-associated domains

Finally, the linkage map was constructed using these five markers, SNP *AX-94542331*, PLUG marker *TNAC1674* and SSRs *Tag-SSR10*, *Tag-SSR12* and *Tag-SSR14*. KASP marker *AX-94,542,331* was 3.28 cM away from *Lr*^*sp3603*^ and 6.62 cM farther from *Yr*^*sp3603*^. PLUG marker *TNAC1674* was mapped distally at 9.37 cM and 19.27 cM from *Lr*^*sp3603*^ and *Yr*^*sp3603*^, respectively. *Tag-SSR12* and *Tag-SSR14* were co-segregating and mapped closest to *Lr*^*sp3603*^ at 2.42 cM distance but 12.32 cM away from *Yr*^*sp3603*^. *Tag-SSR10* was positioned at 0.32 cM from the other two co-segregating SSRs but was mapped 2.81 cM away from the *Lr*^*sp3603*^ (Fig. 4a).

Physical positions of markers *Tag-SSR14* and *AX-94,542,331*, closely linked with *Lr*^*sp3603*^, delimited the gene within ∼ 1 Megabase pairs (Mbp) (Fig. 4b). Annotation of this region revealed a total of 28 gene IDs among which 10 IDs are known to be associated with disease resistance or plant immunity mechanisms (Fig. 4c, Table S4). Three of these, *TraesCS6B03G0098500*, *TraesCS6B03G0098600* and *TraesCS6B03G0101000*, encode for NBS-LRR domains representing a major class of R genes, which play a central role in providing host resistance against various pathogens. On the other hand, the candidate region for *Yr*^*sp3603*^ was not precisely defined due to its location at the distalmost end of the linkage map, with markers positioned only on one side. Functional annotation of ∼ 10 Mbp candidate region around *Yr*^*sp3603*^ was carried out which also identified 25 gene IDs involved in plant defence mechanisms including gene ID *TraesCS6B03G0114500* which encodes typical NBS-LRR resistance like protein (Table S5). Overall, annotation of ∼ 15 Mbp target chromosomal region revealed 10 NBS/LRR encoding gene IDs which can serve as candidates for LR and/or YR resistance (Table S6).

### Confirmation of homoeologous chromosome position of *Lr*^*sp3603*^ and *Yr*^*sp3603*^

To validate the location of *Lr*^*sp3603*^ and *Yr*^*sp3603*^ on chromosome 6B, all markers forming distinct segregation within mapping population were amplified on nullisomic-tetrasomic (NT) stocks of chromosome 6 (*CS-N6A-T6D, CS-N6B-T6A, CS-N6D-T6A, CS-N6D-T6B*) in Chinese Spring background (Fig. S6). *AX-94542331*, *Tag-SSR10*, *Tag-SSR12* and *Tag-SSR14* amplified a single susceptible allele on all stocks except *CS-N6B-T6A*, lacking a 6B homoeologue. This indicated the markers were from chromosome 6B only and absent on chromosome 6 A or 6D. *TNAC1674* amplified two amplicons of size around 600 bp and 700 bp in *CS-N6A-T6D*, *CS-N6D-T6A* and *CS-N6D-T6B*, but only one allele of ∼ 700 bp in *CS-N6B-T6A* indicating the absent allele has 6B homoeologue-specific binding (Fig. S5a, S6e).

## Discussion

Wheat, an essential nutrition staple for billions of people, takes the worldwide lead in production volume and cultivated area [45]. However, it is facing numerous biotic and abiotic challenges due to faded resilience in cultivated wheat germplasm. Among all stresses, leaf rust and stripe rusts have remained the most important diseases of wheat since ancient times. In India, breeding for rust resistance was initiated in the 1930s by Dr B.P. Pal, and since then, the rapidly evolving fungal pathogens have never let a pause in resistance breeding. Both the rusts have been identified with multiple variants such as in LR, the groups named 12, 77 and 104 have repeatedly evolved, resulting in the breakdown of many important LR resistance genes [36, 46]. Similarly, virulence against important YR resistance genes such as *Yr9, Yr17, Yr18, Yr19, Yr21, Yr22, Yr23, Yr25, Yr27* have been detected in newly evolved pathotypes 238S119, 110S119 and 46S119 of yellow rust [47, 48]. The eroding diversity in cultivated wheat and the evolution of pathogens make it essential to explore the unutilized germplasm to enhance genetic diversity and thus tackle the mainstream problems in wheat [49]. Hence, in this study, we aimed to introduce the LR and YR resistance in wheat from the wild progenitor species *Aegilops speltoides*.

*Ae. speltoides* (SS), the diploid wild relative from the secondary gene pool of wheat, is among the five members of the *Sitopsis* section. It shares close similarities with the B genome of wheat, which originated from a diploid extinct species that diverged from *Ae. speltoides* around 4.49 million years ago [49]. It has proven to be a valuable genetic resource for enhancing crop resilience against many abiotic [50, 51] and most importantly biotic challenges like leaf rust, stem rust, powdery mildew and green bug. Numerous resistance genes like *Lr28, Lr35, Lr36, Lr47, Lr51, Lr65*, and *Lr66* against leaf rust; *Sr32, Sr39, Sr47*, and *Sr54* against stem rust; *Pm1d, Pm12, Pm32*, and *Pm53* against powdery mildew and *Gb5* against green bug have been identified from this species [52, 53].

For this study, we selected one of the LR and YR resistant *Ae. speltoides* accession pau 3603, which was found to be resistant to LR and YR when screened against these rusts in PAU for multiple years. To make this resistance usable, it was introduced into the hexaploid wheat background and a hexaploid introgression line named IL^*sp3603*^ was developed by crossing the wild species first with tetraploid durum wheat as bridging species (to bridge the ploidy gap between diploid *Ae. speltoides* and hexaploid wheat) and then backcrossed F_1_ twice with hexaploid wheat to retain chromosome number 2n = 42 and recover wheat background. Then to characterize and map the transferred rust resistance, we created an F_6_ and F_7_ RIL mapping population by crossing IL^*sp3603*^ with LR and YR susceptible cultivar WL711. The dominant nature of *Lr*^*sp3603*^ and *Yr*^*sp3603*^ resistance genes against both the rusts was evident from the F_1_ being resistant against both rusts. Inheritance studies were conducted over two cropping years in F_6_ and F_7_ generation against prevalent leaf rust (LR) pathotypes, 77−5 (121R63-1), 77−9 (121R60-1) and stripe rust (YR) pathotypes, 46S119 (46E159), 110S119 (110E159), 238S119 (238E159). The single major gene designated as *Lr*^*spelt3603*^ and *Yr*^*spelt3603*^ demonstrated the competence of *Ae. speltoides*-derived resistance genes in providing host protection against predominant pathotypes under Indian conditions.

A revolution in the mapping of valuable genes came with the rise of high-throughput next-generation sequencing platforms [24, 54, 55]. Furthermore, these advancements have enabled the development of high-density SNP arrays, like the cost-effective 35 K SNP array with 35,143 data points. Unlike more expensive platforms, such as the 820K array, this condensed version is ideal for large-scale mapping studies and marker-assisted selection in wheat breeding [56, 57]. In the present study, we integrated BSA with high throughput 35K SNP genotyping for rapid identification of genomic region introgressed from LR and YR resistance donor, *Ae. speltoides*. Independent analysis was carried out for LR and YR resistance, both of which pinpointed chromosome 6BS as the target region. Moreover, the amplification of a diverse set of markers like SSRs and PLUG validated the efficacy of this region for providing rust resistance, thereby proving the productiveness of this approach in mapping useful genes. Genotyping success of SNP arrays has also been demonstrated earlier through mapping of *LrTs276-2* on chromosome 1DS using Affymetrix 35K SNP array [58], *LrP* and *YrP* derived from *Aegilops peregrina* on chr5DS [53] and *Lr81* on chromosome 6A using iSelect 90K SNP array [59].

Mapping of LR and YR resistance genes on chromosome 6BS involved 1 SNP, 1 PLUG and 3 SSR markers. Chromosomal positions of these markers revealed a physical coverage of ∼ 15 Megabase pairs (Mbp) spanning from 15.5 Mbp to 30.1 Mbp. *Lr*^*sp3603*^ was specifically confined within the ∼ 1 Mbp segment (29.2–30.1 Mbp) of this region flanked by markers *Tag-SSR14* and *AX-94542331*. The identified NLR-coding gene IDs, *TraesCS6B03G0098500*, *TraesCS6B03G0098600* and *TraesCS6B03G0101400*, serve as promising candidates for *Lr*^*spelt3603*^.

S genome of *Ae. speltoides* shares maximum sequence identity with the B genome of *T. aestivum* [60]. Yet gene introgressions from *Ae. speltoides* into the A and D genomes have been observed, such as *Lr28* on 4AL [61], *Lr47* on 7AL [62], *Lr65* on 2AS [63], *Lr66* on 3AS [64], *Sr32* on 2DS [32], *Gb5* on 7AL [30] which is likely due to the repetitive and homoeologous nature of the wheat genome. Therefore, to validate the homoeologue identity of genes mapped in the current study, we employed nullisomic-tetrasomic (NT) aneuploid stocks developed originally by E.R. Sears in Chinese Spring background [65–67]. Amplification of all the markers near *Lr*^*sp3603*^ and *Yr*^*sp3603*^ genes (*AX-94542331, Tag-SSR10, Tag-SSR12, TNAC1674* and *Tag-SSR14*) on the Nullitetrasomic stocks *CS-N6A-T6D, CS-N6D-T6A* and *CS-N6D-T6B* and absence of their amplification in CS-N6B-T6A confirmed these markers on 6B homoeologue.

Till date, four designated LR resistance genes have been reported on chromosome 6B viz. *Lr36* from *Aegilops speltoides*, *Lr53* from *Triticum dicoccoides*, *Lr59* from *Aegilops peregrina*, and *Lr61* from durum wheat. *Lr36* is the only gene that has been mapped on chromosome 6BS as well as derived from *Ae. speltoides*. The presence of *Lr36* in the present study was tested by amplifying linked SSR *Xgwm88* [68] which was monomorphic between IL^*sp3603*^ and WL711 indicating that the leaf rust resistance gene mapped in the present study is not *Lr36*. Moreover, pathotypes 77−5 and 77−9 have gained virulence against this gene, while *Lr*^*sp3603*^ resistance mapped in the current study, is effective. *Lr53* is effective against all the prevalent Indian leaf rust pathotypes [69] but has been derived from a different progenitor species, *T. dicoccoides* (AB). Its closely linked marker, *Xcfd1* [12] was monomorphic in the IL^*sp3603*^ and WL711 hence, an unlikely candidate for *Lr*^*sp3603*^. *Lr59*, though on chromosome 6B has been introduced from an entirely different origin i.e. *Ae. peregrina* (UUSS) with S genome originating from *Ae. longissima* [23, 70]. Moreover, SSR *Xgwm518* [71] linked with *Lr59* was monomorphic in IL^*sp3603*^ and WL711, ruling out this gene as a candidate for *Lr*^*sp3603*^. *Lr61* has also been mapped on Chromosome 6BS but has been identified from a non-wild source i.e. Chilean durum wheat cultivar by Herrera-Foessel et al. in 2008 [72].

Besides LR resistance genes, three *Yr* genes, *Yr35* (linked to *Lr53*), *Yr36* and *Yr78* have been reported on chromosome 6B. While *Yr35* and *Yr36* have been reportedly derived from *T. dicoccoides*, *Yr78* has been mapped through validation of QTL identified through GWAS studies [73]. *Yr36* is a high-temperature adult plant stage resistance gene [15] but *Yr*^*sp3603*^ is an all-stage resistance gene. The *Yr78* gene is again an adult plant resistance gene that has been delimited within markers, CDM88 and CDM103 with corresponding physical locations, 107.4 Mbp to 118.6 Mbp according to RefSeq v2.1 [74]. However, in this study, *Yr*^*sp3603*^ provides all-stage resistance and is mapped closer to *AX-94542331* which is physically positioned at 30.1 Mbp, far away from *Yr78*. Therefore, *Yr78* and *Yr*^*sp3603*^ are different genes.

## Conclusion

This study utilised an introgression line IL^*sp3603*^ which was developed through crossing and backcrossing *Aegilops speltoides* with susceptible wheat varieties. IL^*sp3603*^ carrying LR and YR resistance in the background of hexaploid wheat, was subsequently crossed with susceptible wheat WL711 to generate a mapping population suitable for characterising and mapping rust resistance genes. We identified closely linked markers spanning a genetic distance of 12.65 cM, which could be beneficial for marker-assisted transfer of novel LR and YR resistance genes in wheat breeding programmes.

### Electronic supplementary material

Below is the link to the electronic supplementary material.

Supplementary Material 1

## Data Availability

Supporting data is provided within the manuscript or supplementary information files. Further inquiries can be addressed to the corresponding author.
