# Supplementary material for: Mapping of Aegilops speltoides derived leaf rust and stripe rust resistance genes using 35K SNP array
Source: BMC Genom Data. 2024 Jul 15;25:69. doi: 10.1186/s12863-024-01247-5 (PMC11247808; doi:10.1186/s12863-024-01247-5)
Supplement: Supplementary file 1 — Supplementary Material 1 [file 12863_2024_1247_MOESM1_ESM.docx]

**SUPPLEMENTARY MATERIAL**

Figures


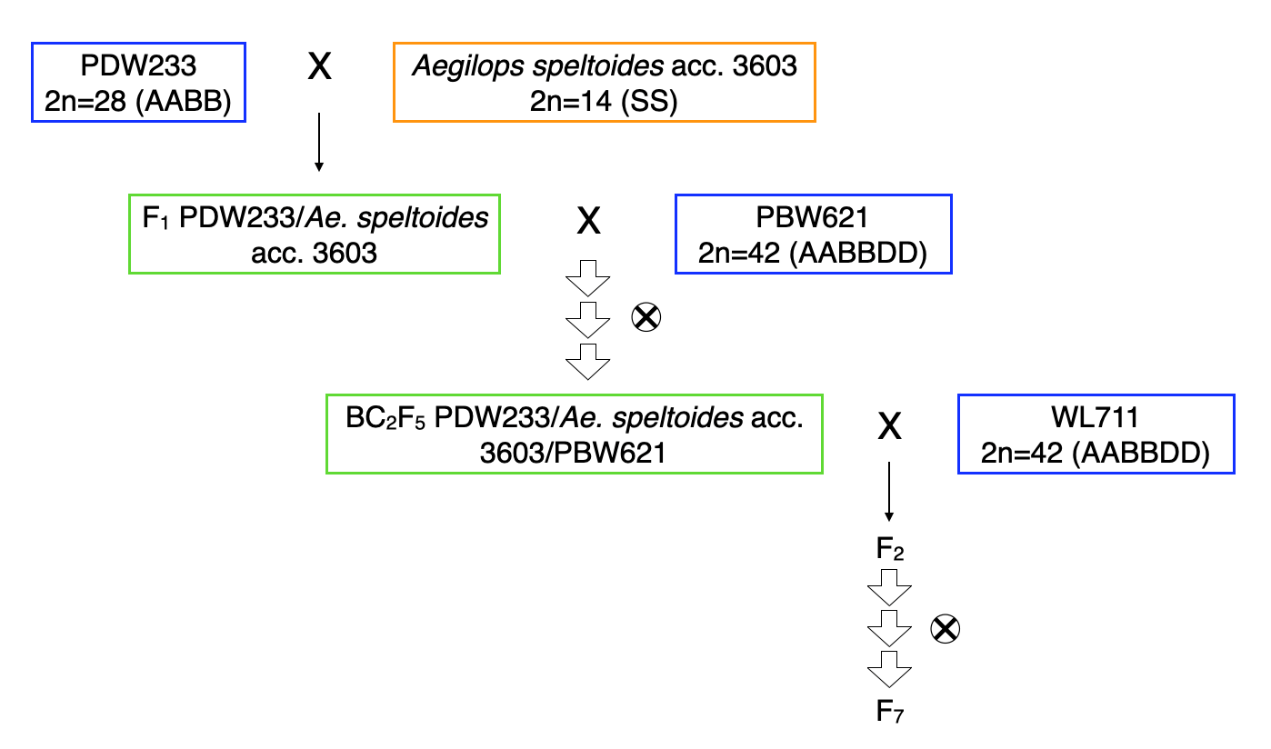


**Fig.S1** Schematic representation of the development of leaf rust and stripe rust resistant introgression line, IL*^sp3603^* from wild progenitor *Aegilops speltoides* acc. pau 3603 in PBW621 background and development of F_7_ mapping population by crossing IL*^sp3603^* with leaf rust and stripe rust susceptible wheat cultivar WL711


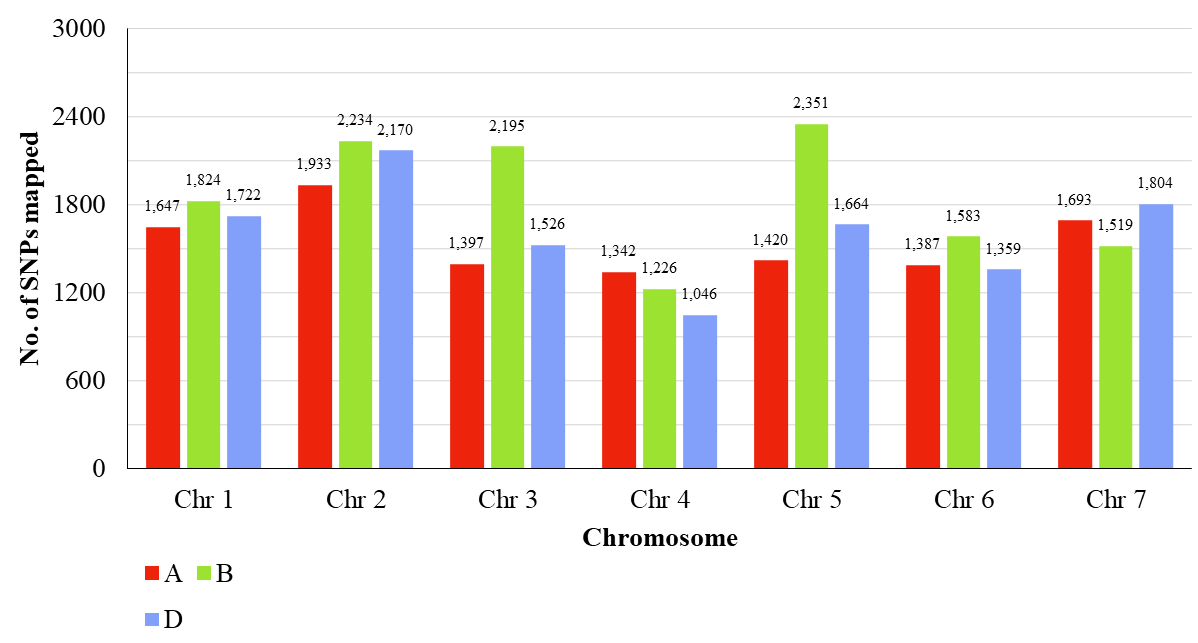
**Fig.S2** Distribution of SNPs mapped across 21 chromosomes in 35K Axiom wheat breeder’s SNP array


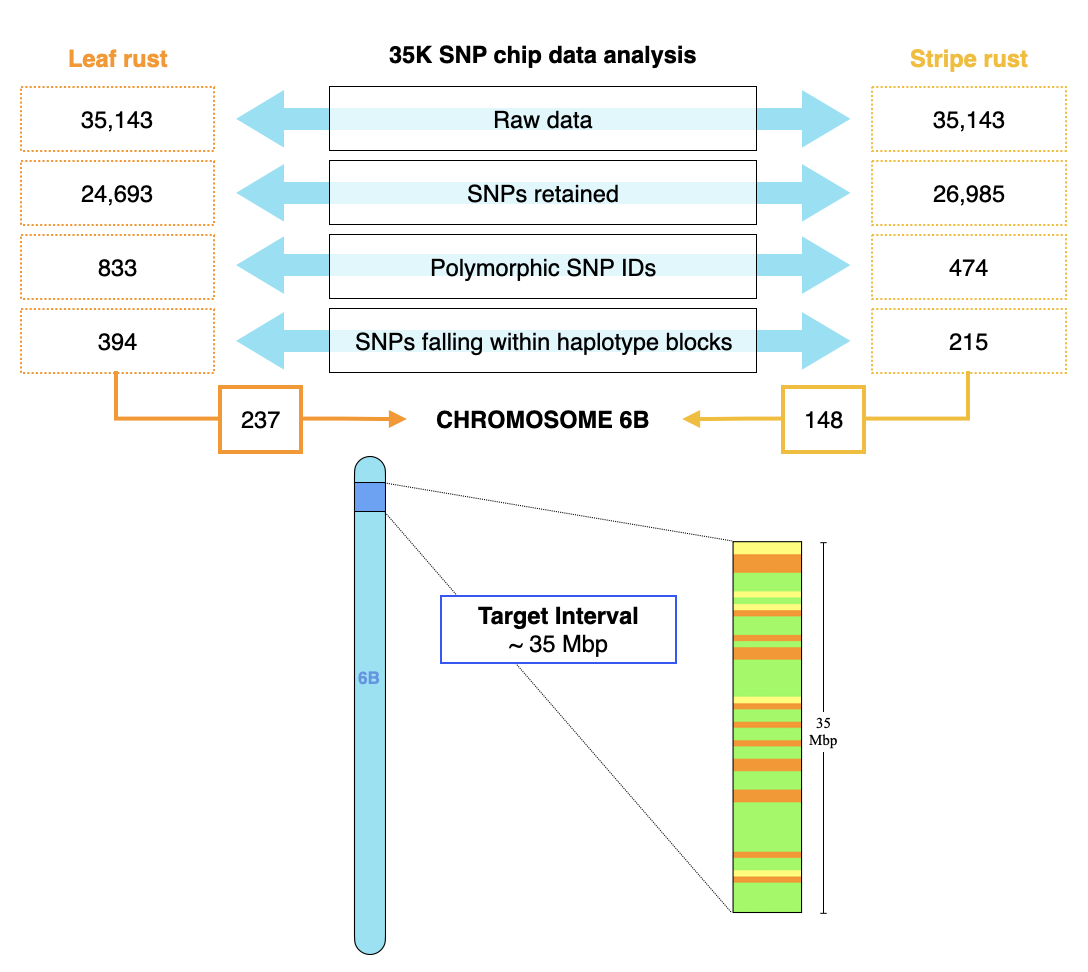


**Fig.S3** Schematic representation of analysis of 35K SNP array data on parental lines (IL*^sp3603^* & WL711) and bulks (for leaf rust and stripe rust) for identification of chromosomal region governing leaf rust and stripe rust resistance (Region highlighted in ORANGE and YELLOW respectively, represents polymorphic SNP IDs identified from individual leaf rust and stripe rust datasets while GREEN denotes the proportion of overlapping SNPs within LR and YR data


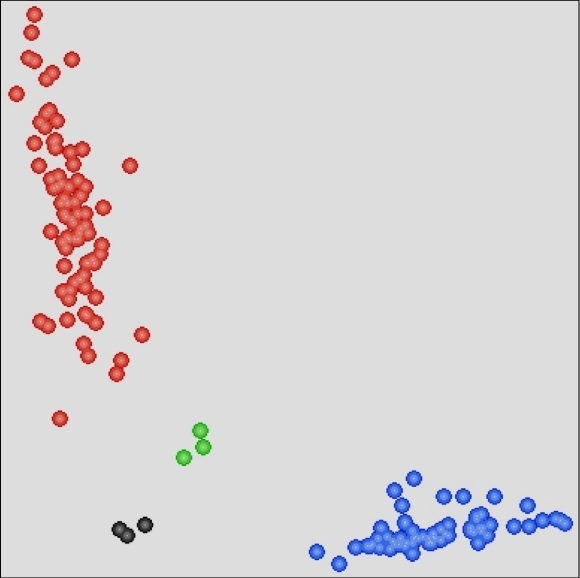


**Fig.S4** Kluster caller output of KASP marker, *AX-94542331* amplified on 152 F_7_ mapping population. RED represents HEX associated ‘T’ allele on y-axis, BLUE represents FAM associated ‘C’ allele on x-axis, GREEN denotes amplification of both the alleles and BLACK represents NTC (No Template Control)


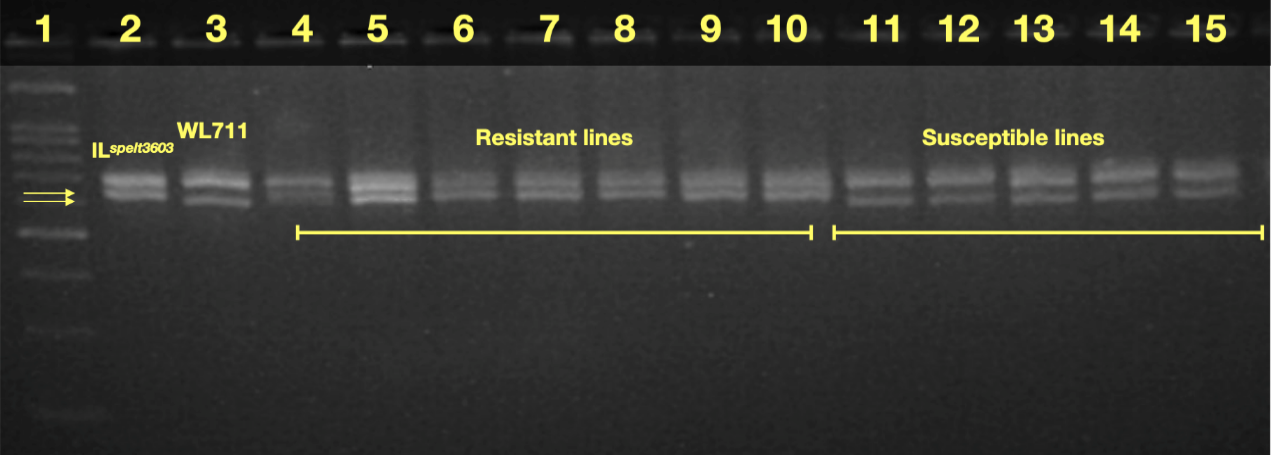


(a)


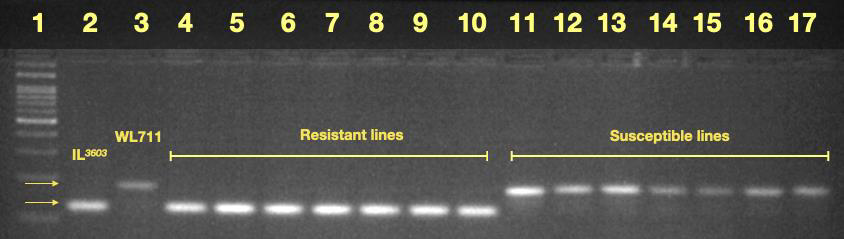


(b)


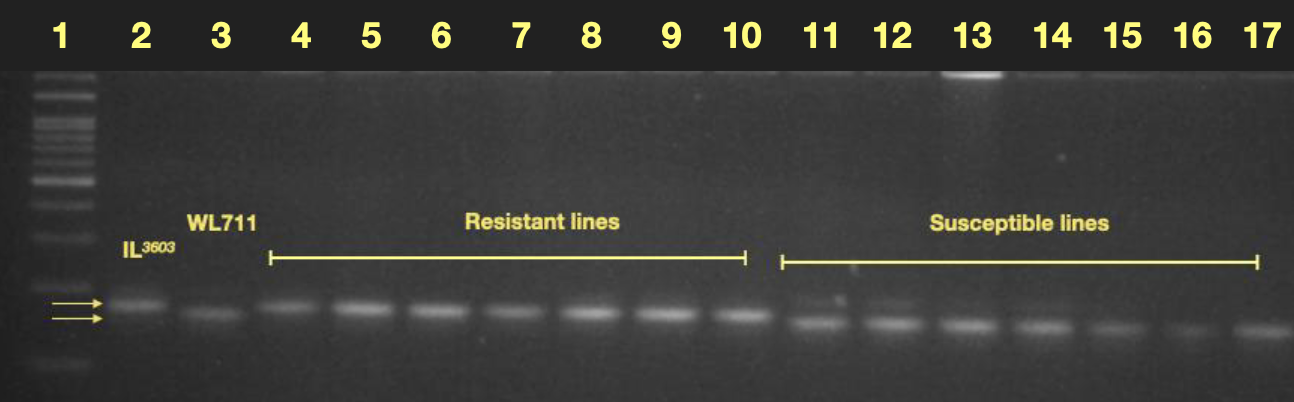


(c)

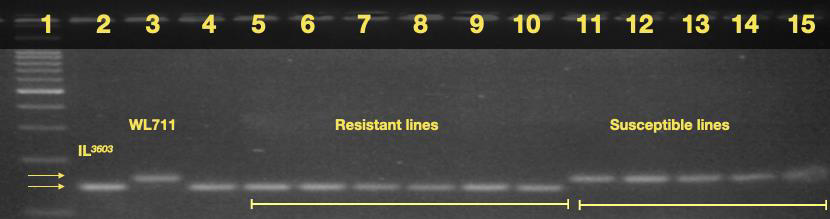


(d)

**Fig.S5** Agarose gel electrophoresis images of amplified products of markers (a) *TNAC1674* (b) *Tag-SSR12* (c) *Tag-SSR14* (d) *Tag-SSR10*


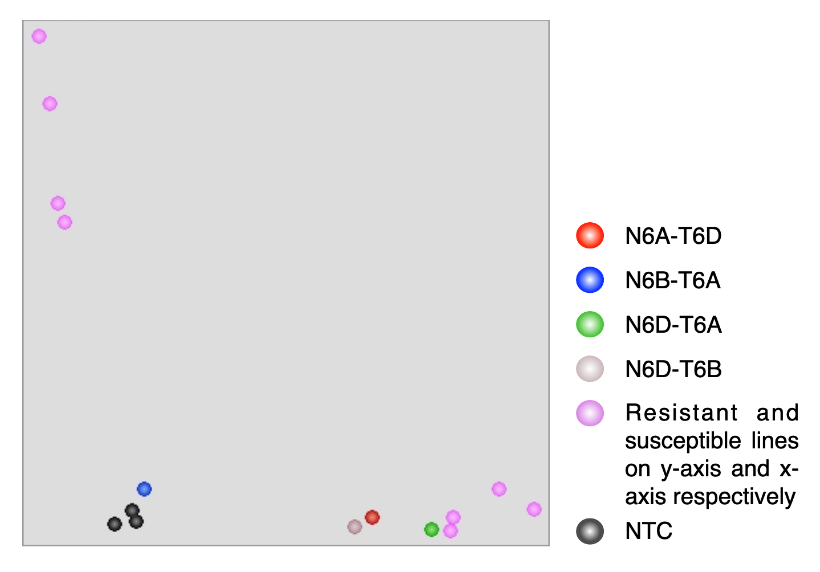


(a)


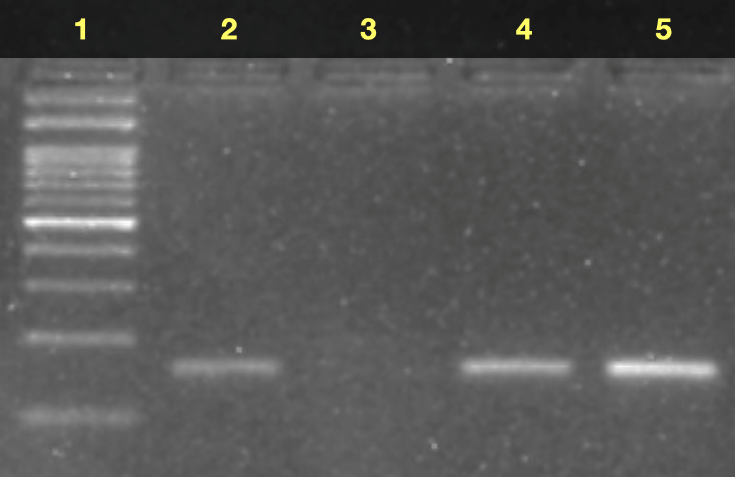


(b)

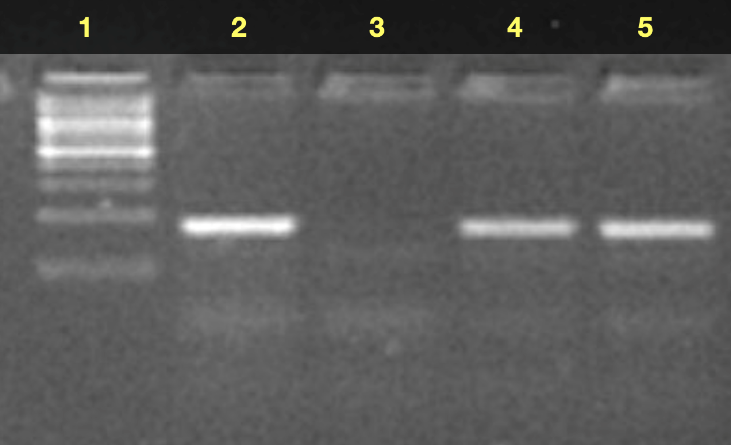


(c)


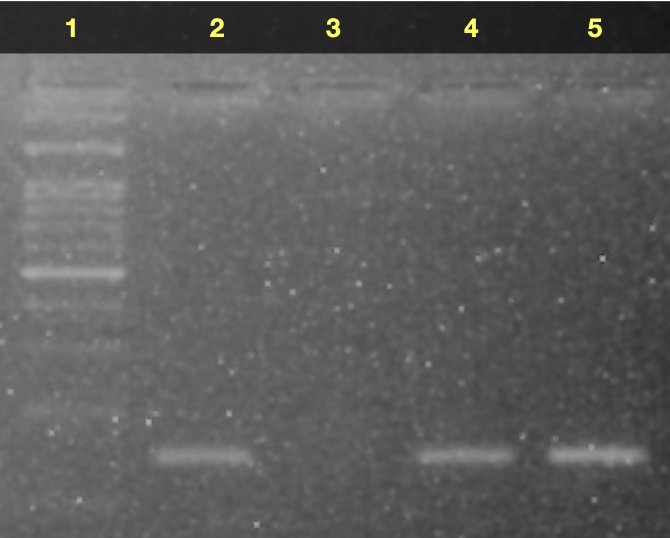


(d)

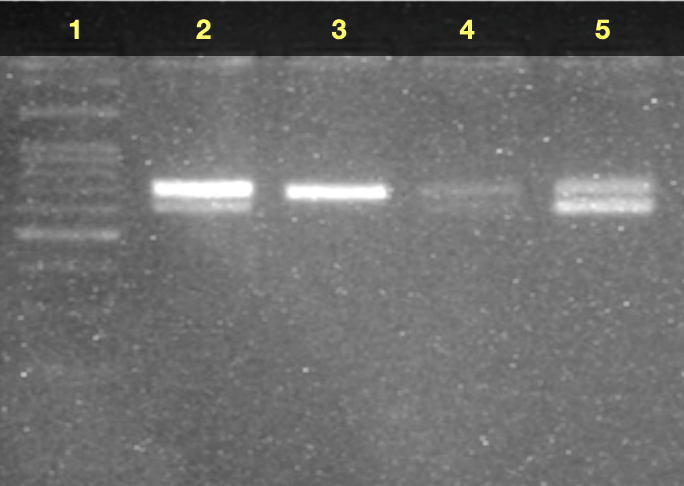


(e)

**Fig.S6** Amplification of markers closely associated with *Lr^sp3603^* and *Yr^sp3603^* genes on nullisomic-tetrasomic deletion stocks of chromosome 6 (a) *AX-94542331* (b) *Tag-SSR10* (c) *Tag-SSR12* (d) *Tag-SSR14* (e) *TNAC1674*. Lane (1) 100 bp ladder, (2) *CS-N6A-T6D*, (3) *CS-N6B-T6A*, (4) *CS-N6D-T6A* (5) *CS-N6D-T6B*

TABLES

**Table S1** Avirulence/virulence formulae of leaf rust and stripe rust pathotypes used in the study

| Pathotype | Avirulence | Virulence |
| --- | --- | --- |
| *Leaf rust* | | |
| 77-5  (121R63-1) | *Lr9, Lr18, Lr19, Lr24, Lr25, Lr28, Lr29, Lr32, Lr39, Lr42, Lr43, Lr45, Lr47* | *Lr1, Lr2a, Lr2c, Lr3, Lr10, Lr11, Lr12, Lr13, Lr14a, Lr14b, Lr14ab, Lr15, Lr16, Lr17a, Lr17b, Lr18, Lr20, Lr21, Lr21a, Lr22b, Lr23, Lr26, Lr27, Lr30, Lr33, Lr34, Lr35, Lr36, Lr37, Lr38, Lr40, Lr44, Lr48, Lr49* |
| 77-9  (121R60-1) | *Lr2a, Lr2b, Lr2c, Lr9, Lr19, Lr24, Lr25, Lr28, Lr32, Lr39, Lr45* | *Lr1, Lr3a, Lr10, Lr11, Lr14a, Lr14b, Lr14ab, Lr15, Lr16, Lr17a, Lr17b, Lr18, Lr20, Lr21, Lr23, Lr26, Lr27 + 31, Lr30, Lr33, Lr36, Lr38, Lr42, Lr44, Lr46, Lr48, Lr49* |
| *Stripe rust* | | |
| 46S119  (46E159) | *Yr1, Yr5, Yr10, Yr11, Yr12, Yr13, Yr14, Yr15, Yr16, Yr24, Yr26, Yrsp, Yrso, Yrsk* | *Yr2, Yr3, Yr4, Yr6, Yr7, Yr8, Yr9, Yr17, Yr18, Yr19, Yr21, Yr22, Yr23, Yr25, YrA, Yrsd, Yrso* |
| 110S119  (110E159) | *Yr1, Yr5, Yr10, Yr13, Yr14, Yr15, Yr16, Yr24, Yr26, Yrsp, Yrsk* | *Yr2, Yr3, Yr4, Yr6, Yr7, Yr8, Yr9, Yr11, Yr12, Yr17, Yr18, Yr19, Yr21, Yr22, Yr23, Yr25, YrA, Yrso* |
| 238S119  (238E159) | *Yr1, Yr4, Yr5, Yr10, Yr13, Yr14, Yr15, Yr16, Yrsk, YrA* | *Yr2, Yr3, Yr6, Yr7, Yr8, Yr9, Yr11, Yr12, Yr17, Yr18, Yr19, Yr21, Yr22, Yr23, Yr24, Yr25, Yr26, Yr27, Yrri, Yrso, Yrsd* |

**Table S2** List of KASP and PLUG markers with their sequences used in this study (FP1- Allele specific Forward Primer 1, FP2- Allele specific Forward Primer 2, RP- Reverse Primer)

| Marker_ID | Primer Sequence |
| --- | --- |
| KASP primers | |
| **AX-94523258** | FP1 5’GAAGGTCGGAGTCAACGGATTaggggaacGtGaacttgaaatG’3  FP2 5’GAAGGTGACCAAGTTCATGCTaggggaacGtGaacttgaaatA’3  RP 5’ggactttttgcaagatacggtC’3 |
| **AX-94728601** | FP1 5’GAAGGTCGGAGTCAACGGATTcgtggttcgacggttccG’3  FP2 5’GAAGGTGACCAAGTTCATGCTcgtggttcgacggttccA’3  RP 5’catccttggagccgataacA’3 |
| **AX-94416516** | FP1 5’GAAGGTCGGAGTCAACGGATTaccaagcagcaaagggaacaaaC’3  FP1 5’GAAGGTGACCAAGTTCATGCTaccaagcagcaaagggaacaaaG’3  RP 5’ccaaactcAgtggaTggtgatTC’3 |
| **AX-94606083** | FP1 5’GAAGGTCGGAGTCAACGGATTgtcctcgagcaCcttggataC’3  FP2 5’GAAGGTGACCAAGTTCATGCTgtcctcgagcaCcttggataT’3  RP 5’taaccccAgtttgaccagcg’3 |
| **AX-94948322** | FP1 5’GAAGGTCGGAGTCAACGGATTgcaccaacagcatcaaaacctC’3  FP2 5’GAAGGTGACCAAGTTCATGCTgcaccaacagcatcaaaacctT’3  RP 5’acttcagagatggcttgagatC’3 |
| **AX-94537931** | FP1 5’GAAGGTCGGAGTCAACGGATTctcactctggaaggtaggctaG’3  FP2 5’GAAGGTGACCAAGTTCATGCTctcactctggaaggtaggctaA’3  RP 5’tgaagcatatggatccattttgG'3 |
| **AX-94996081** | FP1 5’GAAGGTCGGAGTCAACGGATTgggtatgccgaggatgctaatG’3  FP2 5’GAAGGTGACCAAGTTCATGCTgggtatgccgaggatgctaatT’3  RP 5’actggcgaacggatgataca’3 |
| **AX-95228369** | FP1 5’GAAGGTCGGAGTCAACGGATTcccttgtcagtaattgagatctacT’3  FP2 5’GAAGGTGACCAAGTTCATGCTcccttgtcagtaattgagatctacC’3  RP 5’acggtctagttgccagcttt’3 |
| **AX-94728026** | FP1 5’GAAGGTCGGAGTCAACGGATTgtcggcgagacggactcT’3  FP2 5’GAAGGTGACCAAGTTCATGCTgtcggcgagacggactcC’3  RP 5’gaggcccatcccttagcaac’3 |
| **AX-95235777** | FP1 5’GAAGGTCGGAGTCAACGGATTcagtcggcgctgtttgaaA’3  FP2 5’GAAGGTGACCAAGTTCATGCTcagtcggcgctgtttgaaG’3  RP 5’tgtagcaactcgagcttaggc’3 |
| **AX-94871730** | FP1 5’GAAGGTCGGAGTCAACGGATTaacaatacagacaacccaaaatcA’3  FP2 5’GAAGGTGACCAAGTTCATGCTaacaatacagacaacccaaaatcC’3  RP 5’ctgccgtgaacagggaactc’3 |
| **AX-95007535** | FP1 5’GAAGGTCGGAGTCAACGGATTcatgaaaactgtgatgaagcaggT’3  FP2 5’GAAGGTGACCAAGTTCATGCTcatgaaaactgtgatgaagcaggG’3  RP 5’atgcaggcgTaagatgacatgtT’3 |
| **AX-94542331** | FP1 5’GAAGGTCGGAGTCAACGGATTcccacacacacaacaaccaaA’3  FP2 5’GAAGGTGACCAAGTTCATGCTcccacacacacaacaaccaaG’3  RP 5’ctccatgatttctgctgctgc'3 |
| **AX-94599608** | FP1 5’GAAGGTCGGAGTCAACGGATTagcaggcgaagaacatttgatA’3  FP2 5’GAAGGTGACCAAGTTCATGCTagcaggcgaagaacatttgatC’3  RP 5’gctacgacagagtcaccacc’3 |
| **AX-94443986** | FP1 5’GAAGGTCGGAGTCAACGGATTtgaatctctctcacaccagtcaC’3  FP2 5’GAAGGTGACCAAGTTCATGCTtgaatctctctcacaccagtcaT’3  RP 5’ggggagatgaccaccaagtg’3 |
| **AX-94511807** | FP1 5’GAAGGTCGGAGTCAACGGATTagcatgaAgatctagagttcaCG’3  FP2 5’GAAGGTGACCAAGTTCATGCTagcatgaAgatctagagttcaCC’3  RP 5’GctcggtttTggcatTggG'3 |
| **AX-94469037** | FP1 5’GAAGGTCGGAGTCAACGGATTgcccaactcttccaaactgC’3  FP2 5’GAAGGTGACCAAGTTCATGCTgcccaactcttccaaactgT’3  RP 5’tagtacgtgggtttccagcc’3 |
| **AX-94464680** | FP1 5’GAAGGTCGGAGTCAACGGATTtgatccattgaagtcaatcttggC’3  FP2 5’GAAGGTGACCAAGTTCATGCTtgatccattgaagtcaatcttggT’3  RP 5’agttggggcgaaccatatca’3 |
| **AX-95232955** | FP1 5’GAAGGTCGGAGTCAACGGATTtgtatgctgacgtctcccttT’3  FP2 5’GAAGGTGACCAAGTTCATGCTtgtatgctgacgtctcccttG’3  RP 5’tggcacaaagctcagaagc’3 |
| **PLUG primers** | |
| **TNAC1674** | FP 5’CCACCACAGAAGCAGATGAAT’3  RP 5’GCTAGATGGCACACCAAGTG’3 |
| **TNAC1676** | FP 5’ATTGACGCGATTGATAGTGAA’3  RP 5’AGTGGGCTACTGCTTCAGATG’3 |
| **TNAC1677** | FP 5’CCCAAGAGATTTGGCATCATC’3  RP 5’TGGCCATCACCTAAATGAAGA’3 |
| **TNAC1678** | FP 5’AAATCTACTCTGCGAGGTTTGC’3  RP 5’TGGGAAGCGCTTACTATTGTG’3 |
| **TNAC1679** | FP 5’TATTGGCTCAACCAACCATTC’3  RP 5’TTCCAAACCACCCAGTGTGTA’3 |
| **TNAC1683** | FP 5’CTTTCTCTTCTGCAGCTTGGA’3  RP 5’CAGGCAGAACTGGTCAAGAAC’3 |
| **TNAC1685** | FP 5’ATGGATAGCGGAAGCGACTC’3  RP 5’AGCGTTTCCTCCGGTCTT’3 |
| **TNAC1726** | FP 5’CTCAACATCCACGAGTACCAG’3  RP 5’TTTGAAAGTTCCCAATCCAC’3 |

**Table S3** List of gene-based SSR markers, along with corresponding gene IDs and primer sequences used in this study (FP= Forward Primer, RP= Reverse Primer, Tag-SSR = *Triticum aestivum* genic-SSRs)

| Marker_ID | Gene_ID | Primer Sequence |
| --- | --- | --- |
| **Tag-SSR1** | TraesCS6B03G0060400 | FP 5’CTCCTCTCTCTCGCCCTTC’3  RP 5’CGCTCTGGTAGGTAGGAGAA’3 |
| **Tag-SSR2** | TraesCS6B03G0060400 | FP 5’GCCCAATACTCAAGGTACATA’3  RP 5’TATCAATCCACACATCCACA’3 |
| **Tag-SSR3** | TraesCS6B03G0070000 | FP 5’GAGGATCTGGAGGAGCAT’3  RP 5’CGACAGATTAAGTTGCGATT’3 |
| **Tag-SSR4** | TraesCS6B03G0073900 | FP 5’GTGGTTGAGGAGGTAGGTG’3  RP 5’CTAGGCCCATAACCTTCAC’3 |
| **Tag-SSR5** | TraesCS6B03G0079000 | FP 5’TTCGGCTGGTCTATGTTAT’3  RP 5’GCTGATAACCACTAGACACTCAT’3 |
| **Tag-SSR6** | TraesCS6B03G0079000 | FP 5’GTGGCCTTTACAGGAAAATA’3  RP 5’GGAGGGAGTAGAATACAGGAG’3 |
| **Tag-SSR7** | TraesCS6B03G0081900 | FP 5’GATAGCGTCACTGTAATCACC’3  RP 5’GAGTAATTACAGCGCAATGAG’3 |
| **Tag-SSR8** | TraesCS6B03G0081900 | FP 5’GAGCGAACATTATTGACTGAC’3  RP 5’CCCAATTTGGTACTCTCCTAC’3 |
| **Tag-SSR9** | TraesCS6B03G0082100 | FP 5’GTTTGTTACCAGGCTTATGTG’3  RP 5’CTCCGGGTAGAAAAATGTACT’3 |
| **Tag-SSR10** | TraesCS6B03G0085600 | FP 5’GCCTCTTTTTCAGTAGCAATC’3  RP 5’TGGATCATGGATGGACATA’3 |
| **Tag-SSR11** | TraesCS6B03G0090700 | FP 5’GGAATCTAAACCAGGTACGAG’3  RP 5’ATCTTAGAGCCAACATGAGC’3 |
| **Tag-SSR12** | TraesCS6B03G0094100 | FP 5’AAGGTAACCAGGTGCAATACT’3  RP 5’GGTATGGTGTGGTAAGTCAAC’3 |
| **Tag-SSR13** | TraesCS6B03G0098500 | FP 5’ACCTTCAAAATACGATGGAC’3  RP 5’AGAGCCTGAAAAGATGGTACT’3 |
| **Tag-SSR14** | TraesCS6B03G0098500 | FP 5’AATTTCGCTACCACTTCCTAC’3  RP 5’GCTCTGAATTTGTCCCTTCTA’3 |
| **Tag-SSR15** | TraesCS6B03G0100200 | FP 5’CTGCTCACCTGATTCCTG’3  RP 5’GGCCAGATCTAGGAGCTT’3 |

**Table S4** List of candidate LR resistance genes in the mapped region of ~1 Mb along with their genomic positions and functional annotation

| **Gene_ID** | **Position - IWGSC_v2.1 (bp)** | **Description** |
| --- | --- | --- |
| TraesCS6B03G0098500 | 6B:29270237-29284497 | Disease resistance protein (NBS-LRR class) family |
| TraesCS6B03G0098600 | 6B:29374480-29379897 | NBS-LRR-like resistance protein |
| TraesCS6B03G0098700 | 6B:29386349-29390178 | Receptor-like protein kinase |
| TraesCS6B03G0098800 | 6B:29420266-29422557 | Myb/SANT-like DNA-binding domain protein |
| TraesCS6B03G0099300 | 6B:29538643-29540497 | Peroxidase |
| TraesCS6B03G0099400 | 6B:29543998-29545486 | Peroxidase |
| TraesCS6B03G0100000 | 6B:29593903-29595078 | F-box protein |
| TraesCS6B03G0100200 | 6B:29651424-29653069 | F-box family protein |
| TraesCS6B03G0101000 | 6B:29759202-29759678 | Disease resistance protein RPP13 |
| TraesCS6B03G0101400 | 6B:29851611-29852909 | NBS-LRR disease resistance protein |

**Table S5** List of candidate YR resistance genes in the target region of ~10 Mb along with their genomic positions and functional annotation

| **Gene_ID** | **Position - IWGSC_v2.1 (bp)** | **Description** |
| --- | --- | --- |
| TraesCS6B03G0104300 | 6B: 30334622-30337268 | Pentatricopeptide repeat-containing protein |
| TraesCS6B03G0108300 | 6B: 31933447-31934681 | F-box family protein |
| TraesCS6B03G0108600 | 6B: 31956845-31957147 | Cysteine proteinase |
| TraesCS6B03G0108700 | 6B: 31957284-31957919 | Cysteine protease |
| TraesCS6B03G0112200 | 6B: 33254603-33258528 | F-box family protein |
| TraesCS6B03G0114500 | 6B: 33841684-33846519 | NBS-LRR resistance-like protein |
| TraesCS6B03G0116500 | 6B: 34133976-34138237 | Carboxypeptidase |
| TraesCS6B03G0117500 | 6B: 34245597-34252082 | Protein kinase family protein |
| TraesCS6B03G0117600 | 6B: 34261523-34266591 | Protein kinase family protein |
| TraesCS6B03G0117800 | 6B: 34278197-34282596 | Protein kinase family protein |
| TraesCS6B03G0118700 | 6B: 34387785-34389056 | F-box family protein |
| TraesCS6B03G0120000 | 6B: 34864267-34868854 | Receptor-kinase, putative |
| TraesCS6B03G0121400 | 6B: 35663477-35667923 | Receptor-kinase, putative |
| TraesCS6B03G0122700 | 6B: 36310409-36315144 | Kelch repeat-containing protein, f- box |
| TraesCS6B03G0123000 | 6B: 36319288-36321393 | MYB transcription factor |
| TraesCS6B03G0126300 | 6B: 37713358-37715553 | F-box domain containing protein |
| TraesCS6B03G0126400 | 6B: 37716321-37719682 | F-box domain containing protein |
| TraesCS6B03G0127300 | 6B: 38321879-38323129 | Cysteine proteinase |
| TraesCS6B03G0127500 | 6B: 38662346-38663480 | Cysteine proteinase |
| TraesCS6B03G0127700 | 6B: 38832701-38840237 | F-box domain containing protein |
| TraesCS6B03G0128400 | 6B: 39358340-39361591 | F-box family protein |
| TraesCS6B03G0128500 | 6B: 39394192-39397579 | F-box family protein |
| TraesCS6B03G0129000 | 6B: 39510663-39511920 | Cysteine protease, putative |
| TraesCS6B03G0129400 | 6B: 39723891-39725075 | Cysteine proteinase |
| TraesCS6B03G0130500 | 6B: 39848967-39852005 | Receptor-like protein kinase |

**Table S6** List of NBS/LRR encoding genes in the target region of ~15 Mb along with their genomic positions and functional annotation

| **Gene_ID** | **Position - IWGSC_v2.1 (bp)** | **Description** |
| --- | --- | --- |
| TraesCS6B03G0089700 | 6B:26100853-26108055 | NBS-LRR disease resistance protein |
| TraesCS6B03G0090300 | 6B:26236271-26237416 | NBS-LRR-like resistance protein |
| TraesCS6B03G0090700 | 6B:26460649-26468807 | NBS-LRR disease resistance protein |
| TraesCS6B03G0091500 | 6B:26727972-26735445 | Leucine-rich repeat receptor-like protein kinase family protein |
| TraesCS6B03G0093800 | 6B:28167744-28169849 | Leucine-rich repeat receptor-like protein kinase family protein |
| TraesCS6B03G0098500 | 6B:29270237-29284497 | Disease resistance protein (NBS-LRR class) family |
| TraesCS6B03G0098600 | 6B:29374480-29379897 | NBS-LRR-like resistance protein |
| TraesCS6B03G0101000 | 6B:29759202-29759678 | Disease resistance protein RPP13 |
| TraesCS6B03G0101400 | 6B:29851611-29852909 | NBS-LRR disease resistance protein |
| TraesCS6B03G0114500 | 6B: 33841684-33846519 | NBS-LRR resistance-like protein |
